# P-1752. Terbinafine Resistance Among Trichophyton Isolates in the United States

**DOI:** 10.1093/ofid/ofaf695.1923

**Published:** 2026-01-11

**Authors:** Nathan P Wiederhold, Hoja P Patterson, Carmita Sanders, James Mele, Marjorie David, Linh Thuy Phung, Connie Gibas

**Affiliations:** University of Texas Health San Antonio, San Antonio, TX; University of Texas Health San Antonio, San Antonio, TX; UT Health San Antonio, San Antonio, Texas; UT Health San Antonio, San Antonio, Texas; UT Health San Antonio, San Antonio, Texas; UT Health San Antonio, San Antonio, Texas; UT Health San Antonio, San Antonio, Texas

## Abstract

**Background:**

Dermatophytosis is the most common fungal infection worldwide. Outbreaks of terbinafine resistant infections have been reported in parts of India, of which many have been due to a hypervirulent species, *Trichophyton indotineae.* Terbinafine resistant infections, including those caused by this species, have been reported in several countries throughout the world. Here, we report the rate of terbinafine resistance in *Trichophyton* isolates in the United States over a 52-month period.

**Methods:**

Dermatophyte isolates sent to our reference mycology laboratory (Fungus Testing Laboratory, UT Health San Antonio) from at least 25 different states between January 2021 and April 2025 were included. Antifungal susceptibility testing was performed by broth dilution methods according to the CLSI M38 standard. Species identification was performed using phenotypic characteristics and DNA sequence analysis of the ITS and D1/D2 regions and the ß-tubulin gene.

**Results:**

Between January 2021 and April 2015, susceptibility testing was performed against 913 dermatophyte isolates. The most prevalent species was *T. rubrum* (389 isolates), and 77 isolates were identified as *T. indotineae.* Overall, the majority of isolates were inhibited by terbinafine and itraconazole (modal MICs of 0.008 µg/mL and ≤ 0.03 µg/mL, respectively). However, terbinafine resistance (MIC ≥ 0.5 µg/mL) was observed in 165 isolates (18.1%). 72 (93.5%) of the *T. indotineae,* and 61 (15.7%) of *T. rubrum* isolates were terbinafine resistant (MIC range 0.5 - > 2 µg/mL; modal MICs > 2 µg/mL). Elevated itraconazole MICs (≥ 0.5 µg/mL) were observed in 14 of 913 isolates (1.5%), of which 3 were also terbinafine resistant.

**Conclusion:**

Terbinafine resistance is now frequently observed in *Trichophyton* isolates within the U.S. Many of these isolates have been identified as the hypervirulent species *T. indotineae,* which has been responsible for outbreaks of dermatophytosis reported in different parts of the world. However, many of the terbinafine resistant isolates in the U.S. thus far are *T. rubrum.* Continued laboratory and clinical surveillance for antifungal resistant *Trichophyton* infections is warranted.

**Disclosures:**

Nathan P. Wiederhold, PharmD, Basilea: Grant/Research Support|bioMerieux: Grant/Research Support|Bruker: Grant/Research Support|F2G: Grant/Research Support|Mycovia: Grant/Research Support|Scynexis: Grant/Research Support|Sfunga: Grant/Research Support

